# M&A and early investment decisions by digital platforms

**DOI:** 10.1007/s40812-022-00223-3

**Published:** 2022-07-23

**Authors:** Zelda Brutti, Luis E. Rojas

**Affiliations:** 1 Departamento de Economia, Universitat Pompeu Fabra, Edifici Jaume I-Carrer Ramon Trias i Fargas 25-27, 08005 Barcelona, Spain; 2 Barcelona School of Economics, Barcelona, Spain; 3 Institut d’Economia de Barcelona, Barcelona, Spain; 4 Department of Economics and Economic History, Autonomous University of Barcelona, Barcelona, Spain

**Keywords:** M&A, Digital platforms, Platform competition, Consumer welfare, L81, L40, L25

## Abstract

We propose an original theoretical framework that models early investment decisions of digital platform startups and use it to study how merger and acquisition policy affects consumer welfare by shaping such decisions. We formalize the investment options faced by digital platforms into a dual margin: investment in ‘customer engagement technology’, directed towards expanding the user base and in ‘intermediation technology’, directed towards lowering operational costs. Synergies through technological transfer and increased investment incentives in customer engagement explain consumer welfare improvements in the case of M&As occurring between platforms with disjoint user bases. On the other hand, lower competition erodes consumer welfare in the case of allowing M&As between platforms with overlapping user bases. We conclude that M&A policy guidance should depend on the relationship between the incumbent’s and startup’s target users and on the ability of the startup to catch up with the incumbent.

## Introduction

The importance of digital transaction platforms in the global economy has been growing steeply over recent years, with their number more than doubling and their average per capita traffic increasing threefold between 2013 and 2018 across OECD countries (Costa, 2021). Also known as ” digital matchmakers” , digital platforms facilitate exchanges through improved matching of consumers and sellers, reductions in transaction costs and global user reach, creating potentially large economic benefits and increases in market efficiency (Cramer, 2016). The recent Covid-19 pandemic has further boosted the market share of multi-sided online platforms across several sectors, such as retail marketplaces, restaurants and professional services (OECD , 2021).

Successful platforms grow fast^1^ and a remarkable volume of merger and acquisition (M&A) activities is observed in digital platform ecosystems (Motta, 2021; Cabral, 2021; Parker et al., 2021); most of these processes are initiated by large multisided platforms and target young and innovative startups (Gautier, 2021; Argentesi, 2021; Fons-Rosen, 2021). The intensity of M&A operations in this sector has been attracting the attention of academic literature and antitrust authorities alike, given that such activities can enhance efficiency but also be a threat to competition, so that predicting the overall effect on consumer welfare is far from straightforward. The need for well-designed M&A regulations is feeding a lively policy debate on the growing digital platform market.

In this paper, we contribute to the discussion by analyzing how different M&A policy regimes shape the initial investment incentives of platform startups, as these enter an ecosystem featuring an established incumbent. We build an original model that illustrates how the investment decisions of a new platform are influenced by the prospect of being acquired by the established incumbent. This framework allows us to compare consumer surplus in the case in which M&As are allowed and anticipated by the entrant, versus the case in which M&A is restricted. We are able to make statements on the optimal M&A policy and show that this depends on the competitive advantage of the incumbent with respect to the new entrant: when this is large, consumer surplus is maximized when M&A activity is allowed, while when the advantage is small, customers benefit from having M&A restricted.

In our model, customers with heterogeneous tastes across a variety of goods decide whether to shop through a digital platform or by themselves. The platform offers the advantage of a lower cost of searching their most preferred good among those offered by sellers and charges transaction and participation fees in exchange. We focus on the frequent situation of a new platform entering the market and, in its initial development, facing two distinct and complementary investment margins: the first one is investment aimed at expanding its customer base, which we label ‘consumer engagement’ or ‘search technology’ and examples of which may be advertisement, friendliness of the user interface, shopping experience, customer loyalty and similar aspects, all specifically tailored to the variety of products offered by each platform. These investments attract and retain customers by lowering their cost in engaging with the platform, thereby also increasing their private surplus. The second margin is investment aimed at lowering operational costs, which we label ‘operational know-how’ or ‘intermediation technology’ and may be thought of as distribution, warehousing and logistics, payment system management, data processing algorithms and related dimensions. These investments are invisible to platform customers and do not affect their surplus directly, but may do so indirectly as they translate into lower platform fees. The formalization of the investment problem faced by digital platforms into this clear-cut, dual margin is a novel contribution of this paper. In a time following the investment decision, the startup may be acquired by the incumbent platform and the two parties bargain over the acquisition price. If the startup is acquired, technology transfer occurs between the two firms: more specifically, the superior intermediation technology capital of the established incumbent is transferred to the acquired entrant, creating a synergy that produces additional surplus, which is split between the two merging parties. On the other hand, the customer engagement capital that was accumulated by each of the two platforms is not transferable and remains specific to each platform label.^2^

We use this novel framework to explore the effects of allowing or restricting M&A in digital platform ecosystems, distinguishing between two opposite cases: (1) the entrant and the incumbent are not competitors, as they appeal to two separate customer bases; (2) the entrant and the incumbent compete for the same customers. In case (1), an M&A operation has the purpose of creating and splitting new surplus through technology transfer. In case (2), an M&A is a so-called ‘killer acquisition’, whose main objective is to eliminate the new competitor.

In our first main result, we show that in the case of non-competing platforms, a permissive M&A regime shifts the investment incentives of new market entrants towards investments in consumer engagement and away from those in intermediation technology. This is due to the fact that a larger consumer base will increase the entrant’s bargaining power and surplus share in a M&A operation, while own investments in intermediation technology will lose importance due to the incoming technology transfers from the superior incumbent. Along with the value-creation occurred through the technology transfer, the shift in investment towards consumer engagement increases consumer surplus when M&A is allowed.

On the other hand, in our second result we show that in the case of overlapping customer bases, the prospect of an M&A does not shift the investment decisions of the startup across its two margins, although it does affect the overall scale of investment, with the overall effects on consumer surplus being neutral at best, but negative in all those cases in which the level of competitiveness of the startup is such that it would pose a threat to the incumbent. For a startup that poses a threat but is not highly competitive and would be priced out by the incumbent in a no-M&As scenario, the anticipation of the killer acquisition increases investment incentives, with the purpose of enlarging its bargaining power in the M&A operation. The incumbent performs a killer acquisition when this is allowed, whereas under a no-M&A policy it would lower its fees in order to price out the startup, thereby increasing consumer surplus. Conversely, a competitive startup that under a no-M&As regulation would fully benefit from its investment efforts while coexisting and competing with the incumbent will lower its investment scale when M&As are allowed, considering that the perspective of being killer-acquired reduces the scope of its investments to merely improving bargaining power during the negotiation. We show that in such a scenario, allowing for M&As reduces consumer surplus by preventing competition.

In the concluding part of the paper, we extend our analysis to the situation in which the entrant platform has to decide on investment before knowing whether it will be in direct competition for customers with the incumbent or not. Our model reveals that, all things considered, the antitrust policy maximizing consumer surplus depends on how difficult it is for the entrant to catch up with the competitive advantage of the incumbent. It is optimal to allow for M&A when it is very costly for the startup to catch up with the incumbent in terms of technology. This derives from the fact that when M&As are allowed, a not particularly competitive startup knows that both in the case of ending up in non-direct competition and in the case of ending up in direct-competition with the incumbent, investments in consumer engagement will increase its bargaining power and payoff share in the M&A operation, so that it will be incentivized to invest in that dimension and thus increase consumer surplus. If M&As were not allowed, the startup would derive zero payoff from its engagement investments in the direct competition case, since it would be priced out of the market by the incumbent. Additionally, when the gap between incumbent and entrant is large, benefits from technology transfer, which also benefit the consumer through lower fees, are also large. Conversely, it is optimal to restrict platform M&As when it is not very costly for the entrant to catch up with the incumbent. Such a situation, the benefit from technology transfers would not be as large and the consumer would benefit from lower fees in the direct competition case.

The rest of the paper is structured as follows. Section 2 places our contribution among the recent literature on M&A activity on digital markets; Sect. 3 sets up the basic model describing a digital platform market; Sect. 4 explores how M&A policy shapes investment decisions by a new entrant; Sect. 5 illustrates the results obtained for the case of disjoint markets, while Sect. 6 does the same for the case of overlapping customer bases; Sect. 7 provides connections between our model and empirical evidence from the real world; finally, Sect. 8 presents concluding remarks in terms of M&A policy guidance under uncertainty.

## Related literature

Merger policies on digital markets and digital platform ecosystems have been the focus of a recent and growing literature in economics, which highlights that guidance for competition authorities is particularly urgent in this sector. Most importantly, as highlighted in Parker et al. (2021) and Argentesi et al. (2021) among others, analyses that study antitrust policy from an ex-post perspective are at high risk of falling short in the fast-evolving digital platform ecosystem, where large network effects and big data advantage enhance concentration remarkably quickly. On the other hand, Cabral (2021) notes that “firm acquisition may be the simplest path for an incumbent to acquire the technology created by an entrant”: part of his paper, which cautions against the abuse of preemptive merger measures in digital ecosystems, highlights the important role of acquisitions as a form of technology transfer in digital industries and “when the entrant’s technology is a complement with respect to the incumbent’s assets, anticipated acquisition provides a significant innovation incentive”. Along the same lines, Crémer et al. (2019) note how, especially in the digital field, “mergers between established firms and start-ups may frequently bring about substantial synergies and efficiencies: while the start-up may contribute innovative ideas, products and services, the established firm may possess the skills, assets and financial resources needed to further deploy those products and commercialize them.”. Whichever standpoint is adopted, traditional merger policy tools have been considered insufficient to assess competition concerns and to perform proper welfare analysis, thus prompting a set of innovative research on the topic; particularly inspiring is the set of work focusing on how the earliest-stage decisions of new market entrants may be shaped by the regulatory framework.

In a recent paper close to ours, Motta and Peitz (2021) build a reduced-form framework to address the possible anti- and pro-competitive effects of the acquisition of *potential* competitors on the big tech market. They find that whenever the start-up has the ability to pursue its project, the merger will be anti-competitive, since the acquisition then becomes either a “left killer acquisition” or an upgrade with suppressed competition. Their results show that the merger can only be pro-competitive if the start-up would not be able to pursue its project absent the merger and if the incumbent will have an incentive to develop the project after acquiring the start-up. Allowing for mergers may increase the expected benefit from innovation, from the perspective of the startup, so that innovation efforts are stimulated. The work of Bryan and Hovenkamp (2020) is also close to ours, as it focuses on the question on how startup acquisition rules affect innovation incentives and, more specifically, the different technology investment margins of new market entrants. The authors find that allowing for acquisitions biases startups towards improving the types of technology that will improve the leader’s and away from those that would help the follower catching up. A general discouragement of future innovations through a fall in the leader’s willingness to pay for new technologies is also found. In this framework, the market leader acquires startups partially to keep them from competing on similar product qualities (reducing differentiation) and partly to improve its own technology. The main policy insights from this analyses are that no-M&A interventions should be warranted in situations where a highly-dominant incumbent acquires a startup whose technology is competitive and that measures should be taken to prevent dominant firms from systematically acquiring startups and “killing” their competing technology. This last conclusion is shared by Gautier and Lamesch (2021), whose empirical analysis documents that “killer acquisitions” performed by tech giants such as Amazon and Facebook may represent the majority of the M&A operations performed between 2015 and 2017, mostly involving infant startups. Fons-Rosen et al. (2021) quantitatively assess the effects of startup acquisitions on innovation through an endogenous growth model, which they calibrate using a large dataset of acquisitions and firm patents. The authors find that the positive incentives of acquisitions on startup creation are slightly outweighed by a lower own innovation activity by the incumbent and a lower implementation rate of startups’ ideas.

Our paper contributes to the existing literature by proposing a novel theoretical framework tailored to the merger activities among digital intermediation platforms and, despite its simplicity, it is able to deliver predictions on how consumer welfare is shaped on this market depending on whether the M&A is lenient or strict. The impacts on welfare are produced by how the anticipation of being acquired, or the absence thereof, shapes the early-stage investment decisions of infant startups. With respect to the previous recent contributions mentioned earlier, we establish a characterization of the two main investment margins that are specific to digital platforms: investments toward expanding the customer base and those directed at improving daily operations. Beyond their conceptual difference and specificity to the digital platform market, the fundamental difference between the two margins lies in their degree of transferability in case of an acquisition. Note that both of these investment types can be broadly defined as ‘technology’ and can thus encompass and be led back to the interpretations of technology adopted by earlier papers.

## Model setup

There is a unit mass of consumers and firms distributed uniformly on the [0, 1] interval. The location of the consumer characterizes her preferences for goods and the location of the firm characterizes the variety of the good it produces. The utility a consumer $i\in [0,1]$ experiences by consuming *q* units of the good sold by firm $j\in [0,1]$ is given by

$$\begin{aligned} u_{i,j}(q)=q\;\frac{1}{1+d(i,j)}, \end{aligned}$$

where *d*(*i*, *j*) is a distance between *i* and *j* that we assume to be given by $d(i,j)=\epsilon \left| i-j\right|$. This specification represents the idea that a consumer perceives the available goods as differentiated: the location on the goods spectrum of her most preferred variety coincides with her own location on the consumer interval ($j=i$); the further away a good’s variety is with respect to her location, the lower the per-unit utility she experiences. The sensitivity to distance is parametrized by $\epsilon$.^3^

The consumers have a fixed endowment of 1 that they use to consume goods on the market. Consequently total expenditures in the market are constant and equal to 1. Since there is no adjustment margin at the expenditure level, we simplify the preferences by setting them linear in the quantity consumed *q*. Using this specification, we fix the total level of expenditures on the market and focus on how alternative market arrangements affect the consumer surplus.

Firms face a constant marginal production cost, which is standardized to 1. Given that there is a continuum of firms in each location, in a competitive equilibrium all prices are equal to 1.^4^ Consumers have en endowment of 1 that they fully allocate to consumption.

Shopping requires effort by consumers and imposes an utility cost given by *s*. By shopping, the consumer can find a firm that offers her most preferred quality and consequently consumer *i* buys variety *i*. Since her endowment is 1 and the price of all varieties is also 1, then the quantity she can buy is $q=1$. Therefore her utility of shopping labelled as $U^{s}$ is given by

$$\begin{aligned} U^{s}&=u_{i,i}(1)-s=1-s. \end{aligned}$$

There is an incumbent (*I*) digital platform that lowers the search cost of the consumers among the firms located in $[0,j^{I}]$. The platform charges a fee of $\tau ^{I}$ per trade and, therefore, the price that a consumer faces when buying a variety through the platform is $1+\tau ^{I}$. At this price, the quantity that can be acquired with the unit of endowment is $1/(1+\tau ^{I})$.^5^ If the consumer shops through the platform *I*, let the shop effort be equal to zero; however, the consumer can thereby only screen firms in the interval $[0,j^{I}]$. The value of shopping through the platform *I* for consumer *i* is thus given by

$$\begin{aligned} U_{i}^{I}=\max _{j\in [0,j^{I}]}\frac{1/(1+\tau ^{I})}{1+\epsilon \left| i-j\right| }. \end{aligned}$$

It follows that a consumer *i* will prefer to shop through the digital platform *I* rather than shop by herself if $U_{i}^{I}\ge U^{s}$.

The customer base participating in the platform then depends on the fee $\tau ^{I}$. For consumers characterized by locations $i\in [0,j^{I}]$, the platform offers their most preferred variety and consequently they consume variety $j=i$; these consumers decide to participate if

$$\begin{aligned} 1-s\le \frac{1}{1+\tau ^{I}}. \end{aligned}$$

If the fee satisfies the previous inequality strictly, then consumers $i>j^{I}$ could also find it optimal to adhere to the platform and acquire variety $j^{I}$, since that would be their most preferred among the set of options available on the platform. Let $i^{I}\ge j^{I}$ be the consumer that is indifferent between shopping by herself or using the platform ($U_{i^{I}}^{I}=U^{s}$). This consumer’s location is given by

$$\begin{aligned} i^{I}=j^{I}+\frac{1}{\epsilon }\left( \frac{1}{(1-s)(1+\tau ^{I})}-1\right) . \end{aligned}$$

If $i^{I}$ is indifferent between adhering or not to the platform, then any $i<i^{I}$ finds it optimal to adhere. Consequently, we can characterize the customer base of the incumbent ($C^{I}$) as a function of its fee as follows:

$$\begin{aligned} C^{I}(\tau ^{I})={\left\{ \begin{array}{ll} j^{I}+\frac{1}{\epsilon }\left( \frac{1}{(1-s)(1+\tau ^{I})}-1\right) &{} \text {if }1-s\le \frac{1}{1+\tau ^{I}}\\ 0 &{} \text {else.} \end{array}\right. } \end{aligned}$$

**Fig. 1:**
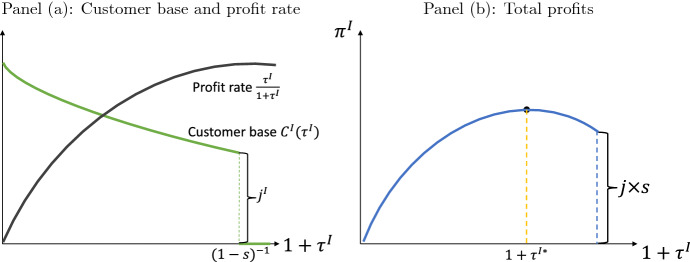
Profit function of the incumbent

The profit function of the incumbent is then given by

$$\begin{aligned} \pi ^{I}=C^{I}(\tau ^{I})\,\frac{\tau ^{I}}{1+\tau ^{I}} \end{aligned}$$

where $\tau ^{I}/(1+\tau ^{I})$ is the profit per consumer the platform obtains.^6^ The larger the fee the larger the profit per consumer but the smaller is the customer base. Figure 1 depicts the customer base, profit per customer as a function of one plus the fee $\left( 1+\tau ^{I}\right)$ in panel (a) and in panel (b) the corresponding profit function. The discontinuous change in the customer base provides the structure to analyze optimal responses at the extensive and intensive margins.

Figure 1 illustrates the case where the maximum profits are reached at an internal solution. However, the optimal fee could also be reached at the corner solution case, where the only customers are $[0,j^{I}]$. The optimal fee $\tau ^{I*}$ that maximizes profits for the incumbent is given by

1 $$\begin{aligned} 1+\tau ^{I*}={\left\{ \begin{array}{ll} \frac{2}{\left( 1+(1-s)\left( 1-\epsilon j^{I}\right) \right) } &{} \text {if }s>\frac{\epsilon j^{I}}{\left( 1+\epsilon j^{I}\right) }\\ \left( 1-s\right) ^{-1} &{} \text {else} \end{array}\right. }. \end{aligned}$$

That is, if the search cost of consumers is large enough, relative to the measure of varieties offered $j^{I}$ and to the preference cost of not buying the most preferred good $\epsilon$, then the platform finds it optimal to set a fee low enough to attract customers in the neighborhood of $j^{I}$.

### Remark 1

Let the size of the incumbent be given by the mass of varieties it offers $j^{I}$. The optimal fee of the incumbent $\tau ^{I*}$ is weakly increasing in its size.

## The new entrant, investment, M&A and competition

Now suppose a new platform enters the market. This platform is characterized by a set of varieties it can offer $J^{E}\subset [0,1]$; an intermediation cost *w*, which can be thought of as the cost of recording and delivering orders, warehousing, data processing, and so forth; a search cost faced by customers $s^{E}$, which can be thought of as the effort or mental cost for the consumer to learn to use and engage with the platform’s interface, as well as any registration costs. For simplicity, we have assumed the intermediation cost and search cost of the incumbent to be equal to zero. This comes without loss of generality, as what is relevant for our next results is the gap between the incumbent and the entrant along these dimensions.

**Investment**

Before entering the market, the entrant has the possibility to invest resources in order to lower the intermediation cost and the search cost. Let $e^{k}$ for $k\in \{s^{E},w\}$ be the resources devoted by the entrant to lower the cost *k*. We assume that the closer the entrant is to the incumbent, the more costly it is to lower these costs further. This is formalized by having that

$$\begin{aligned} \frac{\partial w}{\partial e^{w}}&=-\gamma ^{w}w\\ \frac{\partial s^{E}}{\partial e^{\kappa }}&=-\gamma ^{s}s^{E}. \end{aligned}$$

This structure narrows our approach to a situation where the incumbent is at the ‘technological frontier’ and the entrant can invest to catch up. The specific functional form we have selected is convenient because it allows us to characterize the marginal value of investment as a function of the costs *w* and $s^{E}$ , irrespectively of the investment level and of the level of costs the entrant starts with before investing. The parameters $\gamma ^{k}$ capture how difficult it is for the entrant to catch up with the incumbent along the two cost dimensions.

**M&A and technology transfer**

We consider two policy regimes. One where M&A operations are forbidden and the alternative where they can be implemented freely. A M&A takes place after the investment decisions of the entrant and follows a Nash bargaining protocol, where the surplus of a merged platform is distributed between the two parties involved. The competition alternative represents the outside option for each of the two parties. The new merged entity *M* can set the fees in each of its owned platform labels.

If a M&A occurs, the incumbent can transfer its technology in order to lower the intermediation costs of the entrant. This transfer then achieves $w=0$. Intuitively, this represents the transfer of logistic and technical know-how from the established incumbent to the startup. In such a case, the merged entity *M* can exploit such synergies and the ensuing surplus generated by the merge. We later show that in other cases, a M&A operation could end up in a killer acquisition, in which case intermediation technology is not transferred.

The search cost $s^{E}$ is not altered by the merge. We take $s^{E}$ to be specific to each platform label and the good varieties it offers, so its not fully transferable from another platform, whose experience, interface, customer experience and engagement strategies are specialized in other good varieties. Alternatively, this can be read as a situation in which the entrant cannot perfectly transfer the set of good varieties it can offer $J^{E}\subset [0,1]$, or in other words, its potential customer base to the incumbent label.

In our baseline modeling approach, we adopt the extreme case in which intermediation technology is perfectly transferable between two platform labels, while customer engagement capital cannot be transferred at all. However, what is central for our results is the presence of an asymmetry in the transferability of the two, so that the extreme cases we consider can be relaxed.

**Competition**

If no M&A takes place, the two platforms follow a Stackelberg competition where the incumbent is the leader. A Stackelberg competition framework is particularly suitable to represent the dominant position of the incumbent with respect to the entrant, as the former has the first-mover advantage on the latter. The incumbent decides its fee with full information about its own characteristics and about those of the competitor and can commit to the chosen strategy.^7^ Once the incumbent sets its fee, the entrant decides its optimal fee, also under complete information. This competition structure and the M&A regime is fully anticipated when the entrant has to decide on investment. Finally, consumers observe the fees and decide whether to shop by themselves or adhere to one of the two available platforms.

The varieties the entrant platform can offer, $J^{E}\subset [0,1]$, can give it an advantage with respect to the incumbent. This set describes the niche market the entrant serves, so that the entrant could potentially offer varieties not available on the incumbent platform. We think of these product sets as a fundamental characteristic of digital platform ecosystems. They are meant to capture the feature that, given its unique design, each individual digital platform may be better suited to serve as an intermediary for certain goods rather than others. More specifically, each platform can be thought of as being specialized in revealing the characteristics of a certain set of goods with a high degree of accuracy, to the special benefit of those consumers that most care for that set; examples of such specialized outlets can be found in Sect. 7, where we discuss the outcomes of a survey on investment margins by real-world digital platforms

We take the incumbent to be an Amazon-like platform with a larger variety set than the entrant, who instead serves a niche market. Next, we consider two alternative cases, one in which the entrant niche is disjoint from the incumbent offer and consequently the two platforms do not directly compete for consumers. The other case is the one in which the niche of the entrant is a subset of the incumbent offer, so that the two platform are in direct competition for the same customers.

## Disjoint markets

**Fig. 2:**
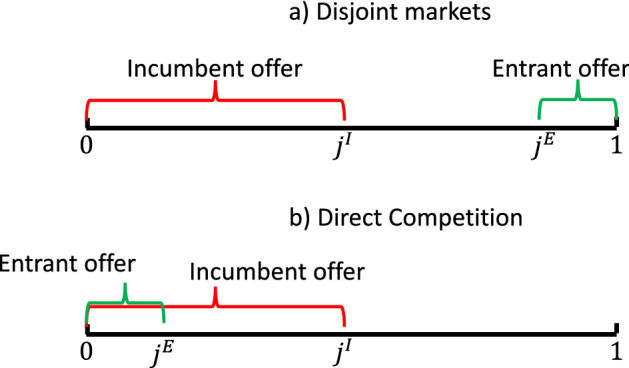
Two extreme cases

Suppose the entrant offers a good variety set $J^{E}=[j^{E},1]$ where $j^{E}>j^{I}$ and the two sets are far enough apart so that the two platforms do not compete directly for the same customers.^8^ Figure 2 presents a graphical representation of this situation. The intuitive outcome in this case is that, due to the absence of direct competition between the two platforms, their technology investments are not changing across different M&A regulations.

For the incumbent, the optimal fee and the customer base are exactly the same as the one shown in the previous section—that is, the decisions of the incumbent do not depend on the M&A regulation in place, nor on the investment decisions of the new entrant.

For the new entrant, the optimal fee and the market it wants to serve are an analogous problem to that of the incumbent, except for the incorporation of the intermediation cost *w* and the search cost $s^{E}$. If an M&A is implemented, the only change will be that $w=0$ by means of the intermediation technology transfer occurring between incumbent and startup.

Given some values of *w* and $s^{E}$, the profit of the new platform is given by

$$\begin{aligned} \pi ^{E}=C^{E}(\tau ^{E})\frac{\tau ^{E}-w}{1+\tau ^{E}}, \end{aligned}$$

where the consumer base is given by the following expression:

2 $$\begin{aligned} C^{E}(\tau ^{E})={\left\{ \begin{array}{ll} \left( 1-j^{E}\right) +\frac{1}{\epsilon }\left( \frac{1}{\left( 1-\left( s-s^{E}\right) \right) (1+\tau ^{E})}-1\right) &{} {\text {if }\frac{1}{1-\left( s-s^{E}\right) }\ge (1+\tau ^{E})}\\ 0 &{} \text {else} \end{array}\right. }. \end{aligned}$$

Note that the entrant’s consumer base (2) follows the same structure as the incumbent’s, with the exception that here profits per customer $\frac{\tau ^{E}-w}{1+\tau ^{E}}$ incorporate the intermediation cost *w* and that the customer base depends on how large the search advantage of the new platform $\left( s-s^{E}\right)$ is, while it depended on *s* for the incumbent.

For a given fee $\tau ^{E}$, the intermediation cost *w* lowers the profit per customer, while the search cost $s^{E}$ lowers the number of customers. Maximizing profits, we find an analogous condition for the optimal fee set by the entrant:

3 $$\begin{aligned} 1+\tau ^{E*}={\left\{ \begin{array}{ll} \frac{2}{\left( 1+w^{E}\right) ^{-1}+\left( 1-\left( s-s^{E}\right) \right) \left( 1-\epsilon \left( 1-j^{E}\right) \right) } &{} \text {if }s-s^{N}>\frac{\left( 1-\left( 1+w^{E}\right) ^{-1}\right) +\epsilon (1-j^{E})}{\left( 1+\epsilon (1-j^{E})\right) }\\ \left( 1-\left( s-s^{E}\right) \right) ^{-1} &{} \text {else} \end{array}\right. } \end{aligned}$$

If the search advantage $s-s^{N}$ is large enough, then the platform sets a fee that allows to attract set of customers that is larger than the set of varieties it offers $(1-j^{E})\le C^{E}(\tau ^{E*})$. The new platform will optimally participate in the market as long as $\tau ^{E*}>w^{E}$ and consequently faces positive profits.^9^

The optimal fee is split in two cases depending on whether the relevant margin for the platform is the intensive or the extensive one. At the intensive margin, the platform is setting a fee at which even consumers that cannot find their most preferred variety in the platform are willing to participate: the platform thus actively attracts customers beyond its variety set. At the extensive margin, the platform sets the highest possible fee at which it will attract only those consumers whose most preferred variety is offered by the platform.

After defining the customer demand elasticity $\upsilon ^{E}=\frac{\partial C^{E}(\tau )}{\partial \left( 1+\tau ^{E}\right) }\frac{(1+\tau ^{E})}{C^{N}(\tau ^{E})}$, we have that $\upsilon ^{E}>1$ if $\epsilon (1-j^{E})<1$ and we refer to this as a high demand elasticity. It is a situation where $\epsilon$ is low enough as to generate a more than proportional increase in demand in response to a fee decrease. The mass of varieties $(1-j^{E})$ shows up in this condition as it scales total demand for a given by $\tau ^{E}$.^10^

### Remark 2

At the intensive margin, a lower intermediation cost *w* implies a lower optimal fee $\tau ^{E*}$. A lower search cost $s^{E}$ implies higher optimal fee when the demand elasticity is high ($\upsilon >1$) and a lower optimal fee otherwise.

At the extensive margin, the intermediation cost *w* has no effect on the fee in any case of participation in the market, while a lower search cost $s^{E}$ implies a higher optimal fee.

The previous remark illustrates the concept that a lower search cost allows the platform to increase fees without losing customers. As a consequence, the platform will generally tend to set higher fees in correspondence to lower search costs. The one exception is a situation of low demand elasticity ($\upsilon <1$), since a decrease in the search cost $s^{N}$ in this case increases the elasticity of demand and consequently makes it optimal to lower fees.^11^

At the extensive margin, since all consumers within the set $[j^{E},1]$ follow the same threshold strategy, the demand elasticity is zero in every point below the fee threshold and is undetermined elsewhere. Therefore, the search cost translates directly into higher fees.

### The value of investment without M&As

We now characterize the marginal value of investment in each scenario, starting from a regime where no mergers and acquisitions take place. The problem of the entrant when deciding the fee is then given by

$$\begin{aligned} \pi ^{E}(w,s^{E})=\max _{\tau ^{E}}C^{E}(\tau ^{E},s^{E})\frac{\tau ^{E}-w}{1+\tau ^{E}} \end{aligned}$$

where $C^{E}(\tau ^{E},s^{E})$ is given in Eq. (2) and here we just make explicit that it also depends directly on the search cost $s^{E}$ but not on the intermediation cost *w*.

Using the Envelope theorem, we can find the marginal value of an investment directed at improving the intermediation cost $(e^{w})$ as:

$$\begin{aligned} \frac{\partial \pi ^{E}(w,s^{E})}{\partial e^{w}}&=\frac{\partial \pi ^{E}(w,s^{E})}{\partial w}\frac{\partial w}{\partial e^{w}} =C^{E}(\tau ^{E*},s^{E})\frac{(-1)}{1+\tau ^{E*}}\frac{\partial w}{\partial e^{w}}\\&\Longrightarrow \frac{\partial \pi ^{E}(w,s^{E})}{\partial e^{w}}&=\frac{C^{E}(\tau ^{E*},s^{E})}{1+\tau ^{E*}}\;\gamma ^{w}w \end{aligned}$$

so that the value of such an investment is proportional to the customer base, given by $C^{E}(\tau ^{E*},s^{E})$, and inversely related to the fee. Once we replace the optimal fee $\tau ^{E*}$ into the previous equation, we get an expression of the marginal value of the investment as a function of the level of the search cost $s^{E}$ and of the intermediation cost *w* as follows:

$$\begin{aligned} \frac{\partial \pi ^{E}}{\partial e^{w}}={\left\{ \begin{array}{ll} G^{w}(w,s^{E})\;\gamma ^{w}w &{} \text {if }s-s^{N}>\frac{\left( 1-\left( 1+w\right) ^{-1}\right) +\epsilon (1-j^{E})}{\left( 1+\epsilon (1-j^{E})\right) }\\ (1-j^{E})\left( 1-\left( s-s^{E}\right) \right) \;\gamma ^{w}w &{} \text {else } \end{array}\right. }, \end{aligned}$$

where $G^{w}(w,s^{E})$ is a continuous function that is decreasing in *w* and finite for $w=0$.^12^

**Fig. 3:**
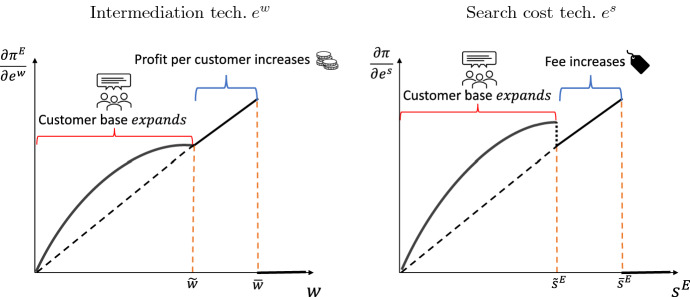
Marginal value of types of investment

Again, the first condition represents the intensive margin and is concave with respect to the cost level *w*, while at the extensive margin it takes the shape of a decreasing and linear function. Figure 3 depicts the relationship between the marginal value and the level of the cost.

Following similar steps, we can find the marginal value of investment on the search cost $e^{s}$ as follows

$$\begin{aligned} \frac{\partial \pi ^{E}}{\partial e^{s}}={\left\{ \begin{array}{ll} G^{s}(w,s^{E})\;\gamma ^{s}s^{E} &{} \text {if }s-s^{N}>\frac{\left( 1-\left( 1+w\right) ^{-1}\right) +\epsilon (1-j^{E})}{\left( 1+\epsilon (1-j^{E})\right) }\\ (1-j^{E})\left( 1+w\right) \;\gamma ^{s}s^{E} &{} \text {else } \end{array}\right. } \end{aligned}$$

where $G^{s}(w,s^{E})$ is a continuous function that is decreasing on $s^{E}$ and finite for $s^{E}=0$.^13^ The marginal value of investment into the search technology $s^{E}$ has a discontinuous jump when the platform moves from the extensive to the intensive margin. The function $G^{s}(w,s^{E})$ evaluated at the threshold is given by $\left( \frac{1}{\epsilon }+(1-j^{E})\right) \left( 1+w\right)$ and consequently the jump is given by $\frac{1}{\epsilon }\left( 1+w\right) \;\gamma ^{s}s^{E}$. This jump captures the feature that lowering the search cost at the extensive margin allows the platform to set a higher fee keeping the same number of customers, while at the intensive margins it also increases the number of customers that participate in the platform for a given fee. Figure 3 panel (b) depicts the relationship between the marginal value and the level of the search cost.

### The value of investment with M&As

Let $\Pi ^{E}$ be the profits of the merged platform on the market segment of the entrant. Since the intermediation technology is fully transferable, after an M&A we have that $w=0$. On the other hand, the search cost $s^{E}$ remains unaltered after the merge. To remind the intuition, this feature reflects the fact that while ‘operational’ technology such as logistic, warehousing and delivery strategies, data processing algorithms and similar know-how can easily be passed between firms, know-how (‘technology’) that relates to customer engagement tends to remain tied to the platform label that created it—i.e. customers won’t easily start using a different platform label just because it is now owned by the same holding entity that owns their preferred platform. Then the profits are given by:

$$\begin{aligned} \Pi ^{E}=C^{E}(T^{E})\frac{T^{E}}{1+T^{E}}, \end{aligned}$$

where $C^{E}(\tau )$ is the same as in Eq. (2) and $T^{E}$ refers to the fee charged by the merged platform on the market segment of *E*. The optimal fee solution follows the same structure as before, just that we set $w=0$.

Importantly, the value of investment in technology for the merged entity is equal to zero $\frac{\partial \Pi ^{E}}{\partial e^{w}}=0$, since technology is transferred from the leading incumbent. On the other hand, the value of investment on the search cost is given by

$$\begin{aligned} \frac{\partial \Pi ^{E}}{\partial e^{s}}={\left\{ \begin{array}{ll} G^{s}(0,s^{E})\;\gamma ^{s}s^{E} &{} \text {if }s-s^{N}>\frac{\epsilon (1-j^{E})}{\left( 1+\epsilon (1-j^{E})\right) }\\ (1-j^{E})\;\gamma ^{s}s^{E} &{} \text {else } \end{array}\right. } \end{aligned}$$

and it presents three differences with respect to the no-M&A scenario.

First, the marginal value of investment at the intensive margin is larger since $G^{s}(0,s^{E})>G^{s}(w,s^{E})$ for $w>0$. The profits the entrant platform can obtain by attracting additional customers through a lower engagement cost are larger without intermediation costs.

Second, the threshold at which the firm moves from the extensive to the intensive margin is higher. Search costs at which the entrant would find itself at the extensive margin under a no-M&A policy warrant intensive-margin participation when M&As are allowed.

**Fig. 4:**
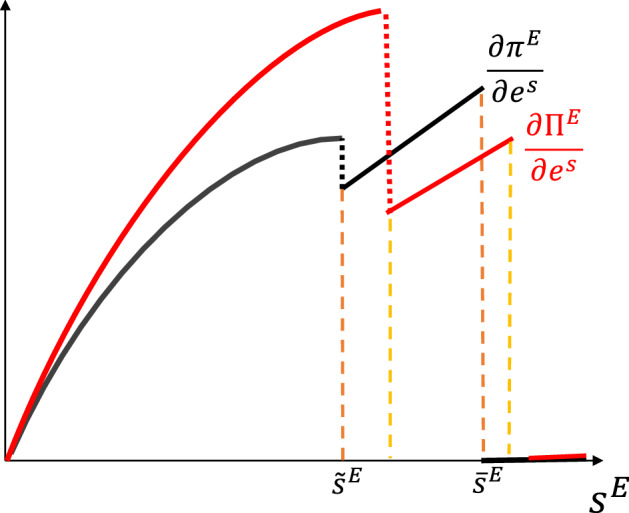
Marginal value of technology investment $e^{w}$ with M&A’s

Third, the marginal value of investment into search cost is lower at the extensive margin. This derives from the fact that the profit rate is less sensitive to the search cost when the intermediation costs are lower—and the profit rates thus already high. These three differences are graphically represented in Fig. 4.

### Bargaining and the investment decision

The surplus created by the M&A is split between the incumbent and the entrant following a Nash bargaining protocol. Letting $\theta \in (0,1)$ denote the bargaining power of the entrant, the entrant’s benefits are given by

$$\begin{aligned} R^{E}&=\theta \left( \Pi ^{E}-\pi ^{E}\right) +\pi ^{E}\\&=\theta \Pi ^{E}+(1-\theta )\pi ^{E}, \end{aligned}$$

where $\left( \Pi ^{E}-\pi ^{E}\right)$ is the surplus obtained by merging and $\pi ^{E}$ is the outside option of the entrant.

If the entrant anticipates the future M&A *before setting its fees,* then the marginal value of investment for the entrant is now given by

$$\begin{aligned} \frac{\partial R^{E}}{\partial e^{s}}&=\theta \frac{\partial \Pi ^{E}}{\partial e^{s}}+(1-\theta )\frac{\partial \pi ^{E}}{\partial e^{s}}\\ \frac{\partial R^{E}}{\partial e^{w}}&=(1-\theta )\frac{\partial \pi ^{E}}{\partial e^{w}} \end{aligned}$$

which, combined with our previous results, delivers that

$$\begin{aligned} \frac{\partial R^{E}}{\partial e^{s}}&\ge \frac{\partial \pi ^{E}}{\partial e^{s}}\\ \frac{\partial R^{E}}{\partial e^{w}}&<\frac{\partial \pi ^{E}}{\partial e^{w}}. \end{aligned}$$

The next proposition follows from the above.

#### Proposition 1

*The prospect of an M* &*A shifts the incentives of investment for the entrant along the two margins. The value of investment into the customer base increases, while the incentives of investing towards lowering the intermediation cost decrease.*

In conclusion, the permissive M&A regulation increases consumer surplus through two different channels: (1) the technology transmission ($w=0$), which implies lower fees and (2) the reshaping of early investment towards lowering the search cost $s^{E}$ in anticipation of the merge.

Note that in this scenario where the platforms are not directly competing, the M&A does not increase the market power of either platform through coordination on fees. This channel will on the other hand become central in the next case we analyze.

## Direct competition^14^

Now suppose that the set of good varieties offered by the entrant is $J^{E}=[0,j^{E}]$, where $j^{E}<j^{I}$. In such a case, the varieties offered by the entrant are a subset of those by the incumbent. Panel (b) in Fig. 2 illustrates this situation. We first focus on a policy regime in which M&As are not allowed.

The platforms compete following a Stackelberg protocol where the incumbent is the leader. Once the entrant observes the fee decided by the incumbent, it decides whether to participate or not. If the entrant participates, then it optimally sets the fee that allows it to attract the consumers $i\in J^{E}$, which are those that find their most preferred good variety offered by the platform and which yield the highest possible profit margin.^15^

For a given fee $\tau$ set by the incumbent, the optimal reaction function of the entrant $\tau ^{E}$ is given by

4 $$\begin{aligned} 1+\tau ^{E}=\frac{1}{\frac{1}{1+\tau ^{I}}+s^{E}} \end{aligned}$$

which is the fee that makes the consumers in the set $[0,j^{E}]$ indifferent between the two platforms. Nevertheless, if the fee implied by the reaction function does not deliver positive profits to the entrant, then the entrant does not participate in the market. The participation condition is then given by

5 $$\begin{aligned} 1+w&<\frac{1}{\frac{1}{1+\tau ^{I}}+s^{E}}\nonumber \\ \Longrightarrow&\left( 1+\tau ^{I}\right) ^{-1}<\left( 1+w\right) ^{-1}-s^{E} \end{aligned}$$

The incumbent, anticipating the reaction of the entrant, decides optimally its fee $\tau ^{I}$ in order to maximize profits. Let $\xi$ serve as a measure of the competitiveness of the entrant and be given by

$$\begin{aligned} \xi =\left( 1+w\right) ^{-1}-s^{E} \end{aligned}$$

that is, let it be decreasing in the search cost and in the intermediation cost. We can use $\xi$ to characterize the optimal pricing decision of the incumbent, which can be summarized into three cases, described as follows:

**Case A: The Entrant is irrelevant**

If the entrant’s competitiveness is lower than the threshold ${ {\xi }}=1-\frac{1}{2}\left( (1-s)\epsilon j+s\right)$, then we have that the fee $\tau ^{I}$ that the incumbent would be setting without competition is already low enough to discourage the entrant from participating in the market—since the participation condition (5) is violated at the optimal price in (1).

In this case, the profits of the entrant are zero and those of the incumbent are exactly the same as if there were no competition.

**Case B: The Incumbent sets the fee to price out the Entrant.**

If the entrant competitiveness is above ${ {\xi }}$ and below a given threshold ${\overline{\xi }}$, then the incumbent finds it optimal to set its fee exactly at the point where the entrant is discouraged from participating, such that

6 $$\begin{aligned} \left( 1+\tau ^{I*}\right) =\frac{1}{\left( 1+w\right) ^{-1}-s^{E}} \end{aligned}$$

and the customer base will be given by

$$\begin{aligned} C^{I}(\tau ^{I*})=j^{I}+\frac{1}{\epsilon }\left( \frac{\left( \left( 1+w\right) ^{-1}-s^{E}\right) }{(1-s)}-1\right) \end{aligned}$$

**Case C: The Entrant takes part of the market**

In this case the competitiveness of the entrant is higher than ${\bar{\xi }}$ and the incumbent finds it optimal to let the entrant take the share of customers that see their most preferred good variety offered on the entrant’s platform. Since the incumbent is losing the customers $[0,j^{E}]$ to the entrant, its own customer base is given by

$$\begin{aligned} C^{I}(\tau ^{I})=\left( j^{I}-j^{E}\right) +\frac{1}{\epsilon }\left( \frac{1}{(1-s)(1+\tau ^{I})}-1\right) \end{aligned}$$

Then it follows that the solution is equivalent to the original problem and given by

7 $$\begin{aligned} 1+\tau ^{I*}=\frac{2}{\left( 1-(1-s)\left( \epsilon \left( j^{I}-j^{E}\right) -1\right) \right) } \end{aligned}$$

which is a lower fee than without competition (Eq. (1)). Note that Remark (1) holds for Cases A and C. The larger the varieties offered by the incumbent $j^{I}$, the bigger the optimal fee $\tau ^{I*}$.

### Remark 3

If the entrant is sufficiently competitive ($\xi >{\underline{\xi }}$), then the consumer surplus benefits from the presence of the entrant.

This remark is a direct consequence of the fact that in cases B and C the incumbent sets a fee $\tau ^{I*}$ that is lower than in case *A*, in which the entrant is irrelevant. The higher surplus comes from the lower fees and the implied higher participation in the platforms. Note that the mechanism behind the lower fees in cases B and C are different. In case B, the lower fees are driven by the policy to price out of the market the entrant. On the other hand, in case C the incumbent loses a market share to the entrant and decides to lower its fee to expand its customer base beyond its offer domain $[j^{E},j^{I}]$.

Our assumption of a Stackelberg competition framework is only relevant for case *C*. In this situation, there exists no equilibrium if we were to assume simultaneous competition. The non-existence of equilibrium at a simultanous game comes from the fact that each platform would have the incentive to cut their fees just below the competitor’s, in order to gain the market share $[0,j^{E}]$; the process would continue up to the point in which the entrant’s fee is low enough $\left( 1+\tau =\left( {\bar{\xi }}+s^{E}\right) ^{-1}\right)$ that the incumbent prefers to concede that segment of the market to the entrant and instead raise its fee for those customers it keeps. Nevertheless, the optimal reaction of the entrant for the larger fee is to raise its own fees too. Consequently, there is no pair of fees for which none of the platforms would like to deviate.

More intuitively, with our setup of Stackelberg competition, the incumbent can commit to setting low fees and price the entrant out of the market when the entrant is not very competitive $\left( \xi <{\bar{\xi }}\right)$, whereas if the entrant is highly competitive, it can commit to accommodate the entrant by renouncing to a market share and not entering into a race-to-the-bottom in fees.

### The value of investment without M&A’s

For Case A, in which the entrant is irrelevant, marginal changes of *w* and $s^{E}$ have no value for the new platform, and neither do they change the value of the incumbent. The marginal value of investment in this range is zero on both margins.

For Case B, the marginal effort by the new platform on $e^{w}$ and $e^{s}$ have no effect on the new platform’s profits but they force the incumbent to set a lower fee and consequently face lower profits. The marginal change in profits of the incumbent as a consequence of changes in the investment margin $e^{k}$ for $k\in \{s,w\}$ is given by

8 $$\begin{aligned} \frac{\partial \pi ^{I}}{\partial e^{k}}=-\left( 2\xi -\left( 1+(1-s)\left( 1-j^{I}\epsilon \right) \right) \right) \frac{1}{(1-s)}\frac{1}{\epsilon }\frac{\partial \xi }{\partial e^{k}}, \end{aligned}$$

where

$$\begin{aligned} \frac{\partial \xi }{\partial e^{s}}&=\gamma ^{s}s^{E}\\ \frac{\partial \xi }{\partial e^{w}}&=\left( 1+w\right) ^{-2}\gamma ^{w}w \end{aligned}$$

and consequently the effect of investment on the incumbent all goes through the increase in competitiveness $\xi$ of the entrant. Since the entrant is still priced out of the market, its profits are zero, as is the marginal value of investment. In this case, marginal changes in *w* and $s^{E}$ are irrelevant for the incumbent. Marginal investment by the entrant does not change the optimal fee of the incumbent, nor its market share.

Now let us move to the most interesting case, Case C, in which the entrant is sufficiently competitive. The profits of the entrant are then given by

$$\begin{aligned} \pi ^{E}=j^{E}\frac{\tau ^{E}-w}{1+\tau ^{E}},, \end{aligned}$$

where $\tau ^{E}$ is given by

$$\begin{aligned} 1+\tau ^{E}&=\frac{1}{\frac{1}{1+\tau ^{I}}+s^{E}} \end{aligned}$$

and consequently we get that the marginal value of investment in the search cost is given by

9 $$\begin{aligned} \frac{\partial \pi ^{E}}{\partial e^{s}}&=j^{E}\left( 1+w\right) \gamma ^{s}s^{E} \end{aligned}$$

and for the investment in the intermediation cost is

10 $$\begin{aligned} \frac{\partial \pi ^{E}}{\partial e^{w}}=j^{E}\left( \frac{1}{1+\tau ^{I*}}+s^{E}\right) \gamma ^{w}w. \end{aligned}$$

Note that the marginal value of investment in the intermediation cost is decreasing in $\tau ^{I*}$ and that this optimal fee is higher as the size of the incumbent is larger (Remark 1), while the marginal value of investment in the search cost does not depend on $\tau ^{I*}$. We therefore derive the following result:

#### Proposition 2

*The size of the incumbent shifts incentives of investment along the two margins at high levels of competitiveness* ($\xi >{\bar{\xi }}$). *The value of investment on the customer base increases, while the incentives to invest on the intermediation cost decrease*.

In region *C* $(\xi >{\bar{\xi }})$, the investment allocation depends on the size of the incumbent, as this captures how high its fees are: the bigger the incumbent, the larger its fees. In this region, it becomes more valuable to invest in the customer base, with the purpose of improving the chances of catching up on fees rather than by lowering the costs.

Finally, note that the profits of the entrant are discontinuous on $\xi$ at the threshold ${\bar{\xi }}$. Here profits jump from zero to a positive level as the entrant moves to participate in the market and the incumbent optimally sets a jump on its fees for $\xi >{\bar{\xi }}$. Consequently, the marginal value of investment at that point is not defined.

### The value of investment with M&As

When platforms compete directly, no added value is created through the existence of the new platform. Since the varieties offered by the entrant are a subset of those offered by the incumbent and since the entrant has higher search and intermediation costs, it is not socially efficient that the entrant operates and engages in investment. Nevertheless, the private incentives of the entrant are to invest, in order to compete with the incumbent and obtain a share of profits that otherwise would be fully appropriated by the incumbent. This competition for profits ends up redistributing rents across platforms but also increases consumer surplus through lower fees.

In this context, the only surplus that a M&A can generate for the two platforms is the avoidance of competition. Since the entrant has no technological advantage over the incumbent, the optimal action for the newly created M&A entity is to stop operating the new platform. The M&A is a thus a so-called “killer acquisition”. This implies that for the merged entity the marginal value of investment is zero.

$$\begin{aligned} \frac{\partial \pi ^{M \& A}}{\partial e^{k}}=0,\quad k\in \{s^{E},w\} \end{aligned}$$

**Fig. 5:**
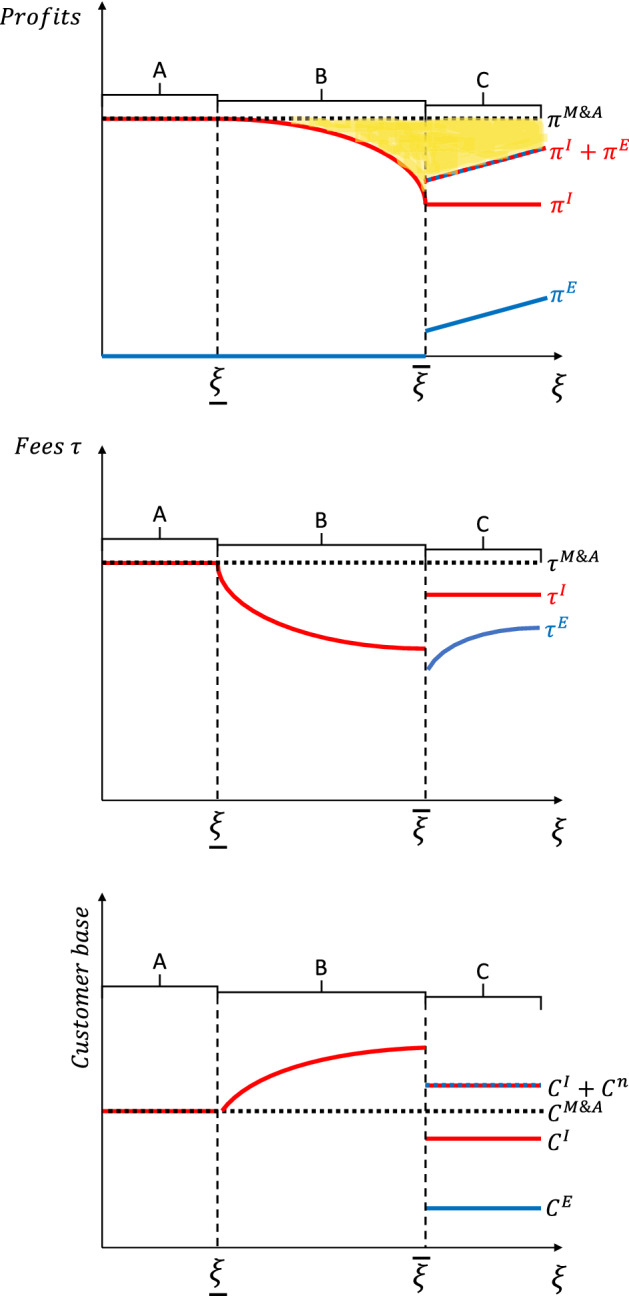
Profits, fees and customer bases for the incumbent, the entrant and the merged platform

Figure 5 depicts the profits of the incumbent and the entrant in case there is no M&A and the profits of the merged platform as a function of the competitiveness of the entrant, $\xi$.

### Bargaining and the investment decision

Despite the early investment decisions of the entrant not increasing the global value of the M&A operation, the entrant still has private incentives to invest, since this affects the outside option of the incumbent (in case B) or of the entrant itself (case C). Recall that in the Nash bargaining protocol, the share of the entrant is given by

$$\begin{aligned} R^{E}&=\theta \left( \Pi ^{E}-\pi ^{E}-\pi ^{I}\right) +\pi ^{E}\\&=\theta \left( \Pi ^{E}-\pi ^{I}\right) +(1-\theta )\pi ^{E} \end{aligned}$$

The shaded region in Fig. 5 depicts the M&A surplus $\left( \Pi ^{E}-\pi ^{E}-\pi ^{I}\right)$. The marginal value of investment is then given by

$$\begin{aligned} \frac{\partial R^{E}}{\partial e^{k}}={\left\{ \begin{array}{ll} 0 &{} \text {if }\xi \le {\underline{\xi }}\\ \theta \left( -\frac{\partial \pi ^{I}}{\partial e^{k}}\right) &{} \text {if }\xi \in [{\underline{\xi }},{\bar{\xi }}]\\ (1-\theta )\frac{\partial \pi ^{E}}{\partial e^{k}} &{} \text {else} \end{array}\right. } \end{aligned}$$

for $k\in \{s^{E},w\}$ where $\frac{\partial \pi ^{I}}{\partial e^{k}}$ and $\frac{\partial \pi ^{E}}{\partial e^{k}}$ are given in Eqs. (9)–(11). So even if the entrant faces a killer acquisition, it has incentives to invest to improve its bargaining position in the M&A operation. In the case of low-competitiveness, its position is improved by weakening the outside option of the incumbent, as the latter would have to sacrifice profits to drive the entrant out of the market. In the case of high-competitiveness, the entrant improves its position by increasing its own outside option through higher profit margins in case of no M&A.

Compared to the regime where M&As are not allowed, we see that the difference is that now the entrant has a positive marginal value of investment in region B, all scaled by the parameter $\theta$; on the other hand, the incentives to invest in region *C* are scaled down by $(1-\theta )$. However, these results refer to the overall level of investment and do not shift the incentives to invest from one margin ($s^{E}$ or *w*) to the other. Therefore we obtain the following result:

#### Proposition 3

*The prospect of an M* &*A does not shift the incentives of investment by startup platforms along the two margins. It does affect the optimal scale of investment of a startup platform, increasing such incentives in low-competitiveness situations and lowering it in high-competitiveness situations.*

## Links with real-world dynamics

Before delivering some concluding remarks on M&A policy guidance that can be drawn from the model we proposed, in this section we provide further insights on the connections existing between our theoretical framework and real-world dynamics.

**The dual investment margin**

The first aspect we provide empirical support for is the type of investments that e-commerce platforms carry out in their day-to-day business. As a reminder, a novel contribution of our theoretical framework has been to propose a simplification of platforms’ investment margins into two main areas, one aimed at expanding the customer base and the other aimed at lowering operational costs. These two areas differ from each other in their degree of ‘visibility’ to the customer (the results of the former have a positive impact on customer experience on the platform, the latter is mostly invisible to users) and in their degree of transferability in the case of an M&A operation (the former produces output that is attached and specific to the platform, therefore harder to transfer to another company, while the latter produces easily-transferable technology and know-how). We can find empirical support for this feature of the model in a recent survey carried out by a well-known digital consulting firm among Italian companies and Italian branches of multinational companies whose official classification of main business activity is e-commerce retail. The sample includes prominent digital retail platforms such as Amazon and Shopify (multi-sector); Interflora (flowers); Emilia Food Love, Mondo del Vino and Negozio del Vino (food and drinks); laFeltrinelli (books); Yumibio (biological cosmetics); Storeden and Asendia (e-commerce intermediation). Note that many of these are real-world examples of platforms that have chosen to focus on a given market niche, which might or might not overlap with the range of products offered on larger platforms such as Amazon, but on which they are able to provide highly specialized information to customers—just as described in our model setup in Sect. 4.

**Fig. 6:**
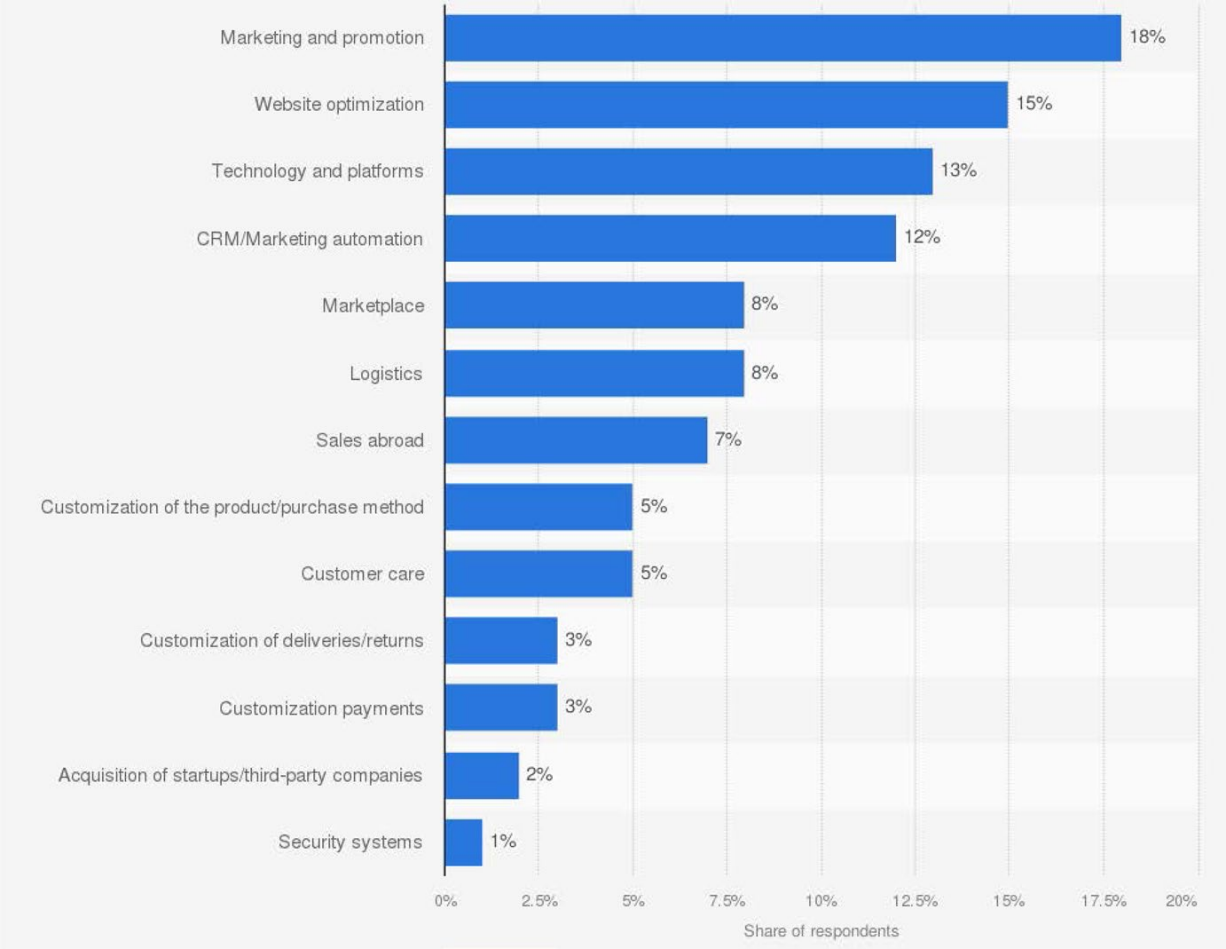
Distribution of short-term investments in the e-commerce sector in Italy, 2021 *Source*: Casaleggio Associati “*E-commerce in Italy 2021*”; $\circledR$Statista 2022.

Figure 6 shows the distribution of responses relating to short-term investments margins envisioned by the interviewees.

Most of the thirteen investment items listed by these real-world digital retail platforms can be smoothly accommodated into one of the two investment areas we work with in our model. Our first area (customer base expansion/customer experience) would comfortably incorporate the items of *Marketing and Promotion*, *Website optimization*, *Customer Relationship Management/Marketing automation*, *Customization of the product/purchase method* and *Customer care*. Our second area (operational technology) would instead absorb the items of *Technology and platforms*, *Logistics* and *Security systems*. There are a few items that might share elements of both areas, so that a finer level of detail on the investment type would be needed for classification (*Customization of deliveries/returns*, *Customization payments*). Finally, there are a couple of items that are harder to classify *(Sales abroad* and *Acquisition of startups/third-party companies*) and which arguably go beyond the scope of our model anyway, given that these appear to be relevant to more established market players, rather than to the world of startups. Under the assumption that the investment menu of platform startups does not differ drastically from the one of their more established counterparts represented in this survey, one can conclude that this data overall supports our claim to have provided a simplified but real-world-like representation of the most relevant decision margins of digital matchmakers.^16^

To complete the mechanism presented in our model, one would wish to additionally be able to provide empirical support to the fact that platform startups invest strategically, once taken into account the M&A regulation and M&A trends surrounding them—and, more specifically, modify their investment margins according to the outlook of being acquired by an established platform along their lifecycle. The fact that the direction of R&D investments should be shaped by M&A has been established in several theoretical papers (among which Bryan and Hovenkamp (2020) and Cabral (2021), see the literature review in Sect. 2), but specific empirical evidence on this point is unfortunately quite hard to come across, as detailed data on investment breakdowns and M&A policy changes is needed.

**Killer acquisitions**

Recent work by Gautier and Lamesch (2021) on M&A operations carried out by the leading digital giants (Google, Amazon, Facebook, Apple and Microsoft—GAFAM) has received attention by several policy institutions formulating M&A guidance, as it represents one of the few systematic empirical analyses on the topic. The paper provides detailed statistics on the acquisitions of smaller digital companies and platforms that the five tech giants, which all have an important platform branch in their activity portfolios, carried out between 2015 and 2017. Among the 175 M&A operations reviewed in Gautier and Lamesch (2021) [G &L henceforth], between 60 and 80% of acquired products were discontinued in their original name and branding: although the authors are unable to distinguish distinguish between pure “killer” acquisitions (in which the product is completely removed after acquisition) and integrations of the acquired technology into the platform ecosystem, they conclude by sounding a note of caution about the potentially very significant “killing” share observed in real world data. In this regard, there is an empirical observation in G &L that fits the empirical predictions of our model very well. Our model predicts that there will be killer acquisitions in the case of overlap between the incumbent’s and the startup’s target audience: indeed G &L find that belonging to the main income segment of the incumbent giant increases the probability of being discontinued after acquisition and their finding is confirmed also in similar empirical work by Argentesi et al. (2021).

**The spotlight on the entrant’s competitiveness**

Our model posits that the startup’s competitiveness is a key feature that determines whether a permissive or restrictive M&A policy will be more beneficial to consumer welfare (see next discussion section, on this point). Empirically, this set of results feeds very well into a recent wave of criticism against the current M&A regulation in the digital sector. In particular, on the basis of the most recent research in the field, several policy institutions have been advising on the importance of leaving behind the traditional ‘sales turnover’ as the main criteria for deciding whether a proposed M&A should be filed for scrutiny by antitrust authorities. In the digital world, due to the extremely rapid scale and network effects characterizing it, startups with low turnover and low current market share but high competitive potential are the very common, so that allowing their unscrutinized acquisition might seriously harm future competition on the market. In response to such concerns, Germany and Austria have recently modified their M&A notification thresholds by including specific reference to the transaction value rather than the turnover as a decision criterion and, more in general, by shifting on the acquirer the burden of proof that the acquisition is pro-competitive.^17^ Our model provides full theoretical support to decisions of this sort, highlighting how the impact that digital platform M&A has on consumer welfare is heterogeneous, depending on competitiveness (and not sales turnover) of the startup. The next section adds some further detail to this point, by drawing some summary conclusions about the policy lessons that one can draw from our setup.

## Concluding remarks: M&A policy with entry under uncertainty

In this final section we use the findings of the novel theoretical framework we have proposed in this paper to draw policy advice for the digital platform market. To do so, we will take a perspective that brings our model another step closer to the reality faced by real-world startups. In particular, what if the entrant platform does not know its precise location on the market *before* deciding on its initial investments? The related question is what would the ideal policy be, if the planner can not tell apart the two cases – no competition or direct competition for customers?

We postulate that before deciding how much to invest and on which margin to do so, the entrant faces a certain degree of uncertainty about how much its own market niche is going to overlap with the one of the incumbent. Our model has illustrated that a central element to approach this discussion is evaluating how costly it is for the entrant to catch up with the incumbent in terms of competitiveness- –a concept we have condensed in $\xi$ and which encompasses both types of the technological fronts that characterize a digital platform, intermediation know-how and consumer engagement capital. Suppose that the entrant’s level of competitiveness is very low before investing and, consequently, if it were to compete directly with the incumbent it would be priced out of the market (would not reach region *C*).

In this case, our results show that allowing to merge can be welfare improving. By allowing for a merge, the entrant is provided with insurance against the risk of direct competition with the incumbent, which would leave the former with zero profits. This insurance device will increase the investment level and thus increase the consumer surplus in the case the entrant actually ends up creating a platform that is able to intermediate different good varieties with respect to the incumbent. Furthermore, in that case the M&A increases the consumer surplus further by allowing the cross-platform synergies to lower the intermediation cost of the entrant and generating subsequent lower fees.

On the other hand, if the incumbent platform is not that costly to catch up, our model predicts that consumer surplus would be increased by an antitrust regimes that bans M&As. Intuitively, if entrants can catch up with the incumbent at low cost then blocking the merge improves welfare—since the welfare losses of the missing synergies in case of disjoint customer bases are compensated by the increase in competition, lower fees and higher consumer participation in case platforms directly compete.

## Appendix A: Direct competition

In this case, the new platform enters in the segment $[0,j^{E}]$ where $j^{E}<j^{I}$. The platforms follow a Stackelberg competition where the incumbent is the leader and the new platform is the follower.

### A.1: Optimal fee of the new platform

For given $\tau ^{I}$ of the incumbent, the problem of the new platform is given by

$$\begin{aligned} \max _{\tau ^{E}}C^{E}(\tau ^{E})\frac{\tau ^{E}-w}{1+\tau ^{E}} \end{aligned}$$

where the customer base $C^{E}(\tau ^{E})$ is given by

$$\begin{aligned} C^{E}(\tau ^{E})={\left\{ \begin{array}{ll} 0 &{} \text {if }\frac{1}{1+\tau ^{I}}>\frac{1}{1+\tau ^{E}}-s^{E}\\ j^{E}+\left( \frac{1}{\frac{1}{1+\tau ^{I}}+s^{E}}\frac{1}{\left( 1+\tau ^{nE}\right) }-1\right) \frac{1}{\epsilon } &{} \text {if }\frac{1}{1+\tau }\le \frac{1}{1+\tau ^{E}}-s^{E} \end{array}\right. } \end{aligned}$$

where the second part comes from the fact that the consumer that is exactly indifferent across the two platforms $i^{E}$ is given by

$$\begin{aligned} \frac{1}{1+\tau ^{I}}&=\frac{1}{\left( 1+\tau ^{E}\right) (1+\epsilon (i^{E}-j^{E}))}-s^{E}\\&\Longrightarrow i^{E} =j^{E}+\left( \frac{1}{\frac{1}{1+\tau ^{I}}+s^{E}}\frac{1}{\left( 1+\tau ^{E}\right) }-1\right) \frac{1}{\epsilon } \end{aligned}$$

The new platform will participate in the market only if it selects a fee that satisfies $\frac{1}{1+\tau ^{I}}\le \frac{1}{1+\tau ^{E}}-s^{E}$, or equivalently

$$\begin{aligned} 1+\tau ^{E}\le \frac{1}{\frac{1}{1+\tau ^{I}}+s^{E}} \end{aligned}$$

and a fee that low can only be optimal if it covers the intermediation cost *w*. So, to ensure participation of the entrant, the following condition has to hold

11 $$\begin{aligned} 1+w<\frac{1}{\frac{1}{1+\tau ^{I}}+s^{E}} \end{aligned}$$

so in case of zero profits the firm does not participate.

We will now focus on the case where the new platform captures only $j^{E}$ of the market. In this case, the fee by the new platform is given by

$$\begin{aligned} 1+\tau ^{E}=\frac{1}{\frac{1}{1+\tau ^{I}}+s^{E}} \end{aligned}$$

for this to be the optimal fee, then the solution is a corner solution. This will indeed be the case if

$$\begin{aligned} \left( 1+\tau ^{I}\right)&\le \frac{1}{\frac{1}{\left( 1+w\right) \left( 1+\epsilon j^{E}\right) }-s^{E}} \end{aligned}$$

and below we establish the condition under which the above holds.

### A.2: Optimal decision of the incumbent

The optimal decision faced by the incumbent is now to set the fee to maximize profits. The customer base of the incumbent is given by $[i^{E},i^{I}]$, where each of this thresholds are given by

$$\begin{aligned} i^{E}={\left\{ \begin{array}{ll} \frac{1}{2}\left( j^{E}+\frac{1}{\epsilon }\left( \frac{\left( \left( 1+w\right) ^{-1}\right) }{\frac{1}{1+\tau ^{I}}+s^{E}}-1\right) \right) &{} \text {if }1+\tau ^{I}>\frac{1}{\left( 1+w\right) ^{-1}-s^{E}}\\ 0 &{} \text {otherwise} \end{array}\right. } \end{aligned}$$

and $i^{I}$ is equivalent to the no-competition case

$$\begin{aligned} i^{I}={\left\{ \begin{array}{ll} j^{I}+\frac{1}{\epsilon }\left( \frac{1}{(1-s)(1+\tau ^{I})}-1\right) &{} \text {if }(1+\tau ^{I})\le \frac{1}{(1-s)}\\ 0 &{} \text {else} \end{array}\right. } \end{aligned}$$

where we have used the participation condition of the new platform (Eq. (11)). Equivalently, the total consumer base *I* is given by

$$\begin{aligned} C^{I}(\tau ^{I})={\left\{ \begin{array}{ll} j^{I}+\frac{1}{\epsilon }\left( \frac{1}{(1-s)(1+\tau ^{I})}-1\right) &{} \text {if }1+\tau ^{I}\le \frac{1}{\left( 1+w\right) ^{-1}-s^{E}}\\ \left( j^{I}-j^{E}\right) +\frac{1}{\epsilon }\left( \frac{1}{(1-s)(1+\tau ^{I})}-1\right) &{} \text {if }1+\tau ^{I}\in \left( \frac{1}{\left( 1+w\right) ^{-1}-s^{E}},\frac{1}{\left( 1+w\right) ^{-1}\left( 1+\epsilon j^{E}\right) ^{-1}-s^{E}}\right] \\ j^{I}+\frac{1}{\epsilon }\left( \frac{1}{(1-s)(1+\tau ^{I})}-1\right) -\frac{1}{2}\left( j^{E}+\frac{1}{\epsilon }\left( \frac{\left( \left( 1+w\right) ^{-1}\right) }{\frac{1}{1+\tau ^{I}}+s^{E}}-1\right) \right) &{} \text {if }1+\tau ^{I}\in \left( \frac{1}{\left( 1+w\right) ^{-1}\left( 1+\epsilon j^{E}\right) ^{-1}-s^{E}},\frac{1}{1-\left( s-s^{E}\right) }\right] \\ 0 &{} \text {otherwise} \end{array}\right. } \end{aligned}$$

We abstract from the third case, in which the entrant expands its customer base beyond $[0,j^{E}]$. A condition that rules out this case is

$$\begin{aligned} \frac{1}{\left( 1+w\right) ^{-1}\left( 1+\epsilon j^{E}\right) ^{-1}-s^{E}}&>\frac{1}{1-\left( s-s^{E}\right) } \end{aligned}$$

#### A.2.1: Case A, the entrant is irrelevant

In this case the optimal $\tau ^{I*}$ without competition is such that

$$\begin{aligned} 1+\tau ^{I*}<\frac{1}{\left( 1+w\right) ^{-1}-s^{E}} \end{aligned}$$

In this situation, the decision of the optimal fee is not affected by the existence of the new platform, since the optimal monopoly fee is already low enough to exclude participation of the new platform.

$$\begin{aligned} \frac{2}{\left( 1-(1-s)\left( \epsilon j^{I}-1\right) \right) }&<\frac{1}{\left( 1+w\right) ^{-1}-s^{E}}\\ 1-\frac{1}{2}\left( (1-s)\epsilon j^{I}+s\right)&>\left( 1+w\right) ^{-1}-s^{E} \end{aligned}$$

That is, we need *w* and $s^{E}$ large enough for this to happen. Marginal changes of *w* and $s^{E}$ have no value for the new platform in this case, nor do they decrease the value of the incumbent.

#### A.2.2: Case B, the entrant is priced out

In this case, the fee is set to the lowest possible fee at which the new platform would be able to participate, so that the entrant is priced out by the incumbent. The fee in this case is

$$\begin{aligned} 1+\tau ^{I}=\frac{1}{\left( 1+w\right) ^{-1}-s^{E}} \end{aligned}$$

and the customer base is given by

$$\begin{aligned} C^{I}=j^{I}+\frac{1}{\epsilon }\left( \frac{\left( \left( 1+w\right) ^{-1}-s^{E}\right) }{(1-s)}-1\right) \end{aligned}$$

Here, marginal efforts by the new platform on $e^{w}$ and $e^{s}$ have no effect on the new platform profits but they will decrease the incumbent’s fee and consequently profits. Note that they also have to reduce profits, otherwise the original fee would not be optimal.

The profits of the incumbent are

$$\begin{aligned} \pi ^{I}=C^{I}\frac{\tau ^{I}}{1+\tau ^{I}} \end{aligned}$$

and the effect on the incumbent profits of the new platform effort is given by

$$\begin{aligned} \frac{\partial \pi ^{I}}{\partial e^{w}}&=\frac{\partial C^{I}}{\partial e^{w}}\frac{\tau ^{I}}{1+\tau ^{I}}+C^{I}\frac{\partial \frac{\tau ^{I}}{1+\tau ^{I}}}{\partial e^{w}} \end{aligned}$$

so that

$$\begin{aligned} \frac{\partial C^{I}}{\partial e^{w}}&=\frac{1}{\epsilon }\left( \frac{\left( \left( 1+w\right) ^{-2}\right) }{(1-s)}\right) \gamma ^{w}w\\ \frac{\partial \frac{\tau ^{I}}{1+\tau ^{I}}}{\partial e^{w}}&=\frac{\frac{1}{\left( 1+w\right) ^{-1}-s^{E}}-1}{\frac{1}{\left( 1+w\right) ^{-1}-s^{E}}}=-\left( 1+w\right) ^{-2}\gamma ^{w}w \end{aligned}$$

and

$$\begin{aligned} \frac{\partial \pi ^{I}}{\partial e^{w}}&=\frac{\partial C^{I}}{\partial e^{w}}\frac{\tau ^{I}}{1+\tau ^{I}}+C^{I}\frac{\partial \frac{\tau ^{I}}{1+\tau ^{I}}}{\partial e^{w}}\\&=\frac{1}{\epsilon }\left( \frac{\left( \left( 1+w\right) ^{-2}\right) }{(1-s)}\right) \gamma ^{w}w\frac{\tau ^{I}}{1+\tau ^{I}}-C^{I}\left( 1+w\right) ^{-2}\gamma ^{w}w\\&=\left( \frac{1}{\epsilon }\left( \frac{1}{(1-s)}\right) \frac{\tau ^{I}}{1+\tau ^{I}}-C^{I}\right) \left( 1+w\right) ^{-2}\lambda ^{w}w \end{aligned}$$

Note that the term in parenthesis has to be negative, since otherwise $\tau ^{I}$ would not be optimal.

Replacing $\tau ^{I}$ and $C^{I}$ we get

$$\begin{aligned} \frac{\partial \pi ^{I}}{\partial e^{w}}&=\left( 1+(1-s)\left( 1-j^{I}\epsilon \right) -2\left( \left( 1+w\right) ^{-1}-s^{E}\right) \right) \frac{1}{\epsilon }\frac{\left( 1+2\right) ^{-2}}{(1-s)}\gamma ^{w}w\\ \frac{\partial \pi ^{I}}{\partial e^{c}}&=-\left( 2\left( \left( 1+w\right) ^{-1}-s^{E}\right) -\left( 1+(1-s)\left( 1-j^{I}\epsilon \right) \right) \right) \frac{1}{\epsilon }\frac{\left( 1+w\right) ^{-2}}{(1-s)}\gamma ^{w}w \end{aligned}$$

The marginal effect of effort on the engagement cost is given by

$$\begin{aligned} \frac{\partial \pi ^{I}}{\partial e^{s}}=\frac{\partial C^{I}}{\partial e^{w}}\frac{\tau ^{I}}{1+\tau ^{I}}+C^{I}\frac{\partial \frac{\tau ^{I}}{1+\tau ^{I}}}{\partial e^{w}} \end{aligned}$$

where we obtain

$$\begin{aligned} \frac{\partial C^{I}}{\partial e^{w}}=\frac{1}{\epsilon }\frac{1}{(1-s)}\gamma ^{s}s^{E} \end{aligned}$$

and

$$\begin{aligned} \frac{\partial \frac{\tau ^{I}}{1+\tau ^{I}}}{\partial e^{s}}&=-\gamma ^{s}s^{E} \end{aligned}$$

then we have

$$\begin{aligned} \frac{\partial \pi ^{I}}{\partial e^{s}}&=\frac{1}{\epsilon }\frac{1}{(1-s)}\gamma ^{s}s^{E}\frac{\tau ^{I}}{1+\tau ^{I}}-C^{I}\left( \gamma ^{s}s^{E}\right) \\&=\frac{1}{\epsilon }\gamma ^{s}s^{E}\left( \frac{1}{(1-s)}-\frac{\left( 1+w\right) ^{-1}-s^{E}}{(1-s)}\right) -\left( j^{I}+\frac{1}{\epsilon }\left( \frac{\left( \left( 1+w\right) ^{-1}-s^{E}\right) }{(1-s)}-1\right) \right) \left( \gamma ^{s}s^{E}\right) \\&=-\left( 2\left( \left( 1+w\right) ^{-1}-s^{E}\right) -\left( 1+(1-s)\left( 1-j^{I}\epsilon \right) \right) \right) \frac{1}{(1-s)}\frac{1}{\epsilon }\gamma ^{s}s^{E} \end{aligned}$$

#### A.2.3: Case C, the entrant takes part of the market

This is the case in which the incumbent’s optimal strategy is not to lower the fee so much as to price out the new entrant from the market. Instead, it finds it optimal to share the market.

The optimal fee of the incumbent is given by

$$\begin{aligned} \max _{\tau ^{I}}C^{I}(\tau ^{I})\frac{\tau ^{I}}{1+\tau ^{I}} \end{aligned}$$

where now *I* is given by

$$\begin{aligned} C^{I}(\tau ^{I})&=\left( j^{I}-j^{E}\right) +\frac{1}{\epsilon }\left( \frac{1}{(1-s)(1+\tau ^{I})}-1\right) \end{aligned}$$

Then we have that the solution is equivalent to the original problem and given by

$$\begin{aligned} 1+\tau ^{I*}=\frac{2}{\left( 1-(1-s)\left( \epsilon \left( j^{I}-j^{E}\right) -1\right) \right) } \end{aligned}$$

that is, a lower fee than without competition.

In this case, a higher investment by the new platform has no effect on the outside option of the incumbent but it has an effect on the outside option of the new platform.

The profits of the new platform are then given by

$$\begin{aligned} \pi ^{E}=j^{E}\frac{\tau ^{E}-w}{1+\tau ^{E}} \end{aligned}$$

where $\tau ^{n}$ is given by

$$\begin{aligned} 1+\tau ^{E}&=\frac{1}{\frac{1}{1+\tau ^{I}}+s^{E}}\\&=\frac{1+\tau ^{I}}{1+\left( 1+\tau ^{I}\right) s^{E}} \end{aligned}$$

and consequently we obtain that the marginal value of effort is given by

$$\begin{aligned} \frac{\partial \pi ^{E}}{\partial e^{s}}&=j^{E}\frac{\partial \frac{\frac{1}{\frac{1}{1+\tau ^{I}}+s^{E}}-1-w}{\frac{1}{\frac{1}{1+\tau ^{I}}+s^{E}}}}{\partial e^{s}}\\&=\frac{\partial j^{E}\left( 1-\left( 1+w\right) \left( \frac{1}{1+\tau ^{I}}+s^{E}\right) \right) }{\partial e^{s}}\\&=j^{E}\left( 1+w\right) \gamma ^{s}s^{E} \end{aligned}$$

and for the effort in the cost is

$$\begin{aligned} \frac{\partial \pi ^{E}}{\partial e^{w}}=j^{E}\left( \frac{1}{1+\tau ^{I}}+s^{E}\right) \lambda ^{w}w \end{aligned}$$

The bigger the incumbent, the larger the $\tau$ and the more valuable is investing in the customer engagement cost.

The optimal mix should set up the two costs to be equal and consequently

$$\begin{aligned} \frac{\frac{w}{1+w}}{\frac{s^{E}}{1+s^{E}}}&=\frac{1+s^{E}}{\left( \frac{1}{1+\tau }+\kappa ^{n}\right) }\frac{\gamma ^{s}}{\gamma ^{w}}\\ \frac{1/s^{E}+1}{1/w+1}&=\frac{1+s^{E}}{\left( \frac{1}{1+\tau ^{I}}+s^{E}\right) }\frac{\gamma ^{s}}{\gamma ^{w}} \end{aligned}$$

In other words, the larger $\tau ^{I}$, the more the new platform shifts resources towards $s^{E}$ and the more valuable is to increase the fee.

### A.3: What are the conditions to be in A, B, or C

The profit function of the incumbent has to be continuous. We can use it to find the values that parameters have to satisfy at each threshold.

To be in Case A, we need that

$$\begin{aligned} 1-\frac{1}{2}\left( (1-\kappa )\epsilon j+\kappa \right) >\left( 1+w\right) ^{-1}-s^{E} \end{aligned}$$

and we define the threshold value ${ {\xi }}=1-\frac{1}{2}\left( (1-\kappa )\epsilon j+\kappa \right)$.

Then the profit in case B is given by

$$\begin{aligned} \pi ^{I,B}&=C^{I}\frac{\tau ^{I}}{1+\tau ^{I}}\\&=\left( j^{I}+\frac{1}{\epsilon }\left( \frac{\left( \left( 1+w\right) ^{-1}-s^{E}\right) }{(1-s)}-1\right) \right) \left( 1-\left( \left( 1+w\right) ^{-1}-s^{E}\right) \right) \\&=j^{I}-\frac{1}{\epsilon }+\left( \left( 1+w\right) ^{-1}-s^{E}\right) \frac{1}{\epsilon }\frac{1}{(1-s)}\\&\quad \times \left( 1-\left( \epsilon j^{I}-1\right) (1-s)\right) -\left( \left( 1+w\right) ^{-1}-s^{E}\right) ^{2}\frac{1}{\epsilon }\frac{1}{(1-s)} \end{aligned}$$

and the profit rate in case C is

$$\begin{aligned} \pi ^{I,C}&=C^{I}\frac{\tau ^{I}}{1+\tau ^{I}}\\&=\left( \left( j^{I}-j^{E}\right) +\frac{1}{\epsilon }\left( \frac{1}{(1-s)(1+\tau ^{I})}-1\right) \right) \\&\quad \left( 1-\frac{1}{2}\left( 1-(1-s)\left( \epsilon \left( j^{I}-j^{E}\right) -1\right) \right) \right) \\&=\left( \left( j^{I}-j^{E}\right) +\frac{1}{\epsilon }\left( \frac{\left( 1-(1-s)\left( \epsilon \left( j^{I}-j^{E}\right) -1\right) \right) }{2(1-s)}-1\right) \right) \\&\quad \times \left( 1-\frac{1}{2}\left( 1-(1-s)\left( \epsilon \left( j^{I}-j^{E}\right) -1\right) \right) \right) \\&=\frac{\epsilon (1-s)}{4}\left( \left( j^{I}-j^{E}\right) +\frac{1}{\epsilon }\left( \frac{s}{(1-s)}\right) \right) ^{2} \end{aligned}$$

if $w=0$ and $s^{E}=0$ we get that

$$\begin{aligned} \pi ^{I,B}&=0 \end{aligned}$$

while $\pi ^{I,C}>0$, so there are values of *c* and $\kappa ^{n}$ such that we move from case B to *C*.

Let $\xi =\left( 1+w\right) ^{-1}-s^{E}$ we have that the threshold value ${\bar{\xi }}$ that sets $\pi ^{I,B}=\pi ^{I,C}$ is given by the solution to

$$\begin{aligned}&j^{I}-\frac{1}{\epsilon }+{\bar{\xi }}\frac{1}{\epsilon }\frac{1}{(1-s)}\left( 1-\left( \epsilon j^{I}-1\right) (1-s)\right) \\&\quad -\left( {\bar{\xi }}\right) ^{2}\frac{1}{\epsilon }\frac{1}{(1-s)}=\frac{\epsilon (1-s)}{4}\left( \left( j^{I}-j^{E}\right) +\frac{1}{\epsilon }\left( \frac{s}{(1-s)}\right) \right) ^{2} \end{aligned}$$

On the other hand the profits of the new platform are given by

$$\begin{aligned} \pi ^{n,A}=\pi ^{n,B}=0 \end{aligned}$$

while in case *C* we have

$$\begin{aligned} \pi ^{E,C}=j^{E}(1+w)\left( \left( (1+w)^{-1}-s^{E}\right) -\frac{1}{1+\tau ^{I}}\right) \end{aligned}$$

## Footnotes

1 The causes of this phenomenon have been the object of several recent studies and found to be due to a combination of network effects, economies of scale and to the efficiency of algorithms growing with data volume (Parker et al. 2021; Costa et al. 2021; van der Aalst et al. 2019; Parker, Petropoulos, and Van Alstyne *(forthcoming)*)

2 This assumption can be relaxed to any situation of large asymmetries in the transferability of the two types of capital.

3 Alternatively one could write preferences as $u_{i,j}(q)=q\;\left( 1-d(i,j)\right)$, or using the squared distance, as it is common in Hotelling-type specifications. What is central for our results is that increasing distance lowers the valuation of the variety. The specification we chose is convenient because it leads to closed-form solutions of the optimal fee set by the platform and thus simplifies the analysis.

4 This conclusion would hold also if we were to have a single firm at each location. The constant marginal cost and the demand structure will still imply a unitary price for all varieties.

5 Note in this case we have a demand elasticity of the fee of − 1.

6 Note that $C^{I}(\tau ^{I})$ is the number of customers adhering to the platform, $1/(1+\tau ^{I})$ the number of trades per customer and $\tau ^{I}$ the profit per trade the platform obtains.

7 Most of our results follow through with simultaneous competition. Commitment from the side of the incumbent is only relevant in the case in which platforms compete directly and the entrant is not priced out of the market by the incumbent (that is case C in Sect. 6). We extend this discussion at the end of Sect. 6.

8 Below we provide a formal condition of what “far enough” requires.

9 No participation in case of zero profits is not central for our results. It is a convenient assumption to simplify the setup when the platforms directly compete as the incumbent can price the entrant out of the market by setting a fee equal to the cost of the entrant. Rather than to have to deal with a fee infinitesimally smaller than the cost of the entrant.

10 $\upsilon ^{N}$ is given by

$$\begin{aligned} \upsilon ^{N}=\frac{\left( \left( 1-\left( s-s^{N}\right) \right) (1+\tau ^{N})\right) ^{-1}}{\epsilon (1-j^{N})-1+\left( \left( 1-\left( s-s^{N}\right) \right) (1+\tau ^{N})\right) ^{-1}} \end{aligned}$$

and since $\left( 1-\left( s-s^{N}\right) \right) >0$ has to hold—otherwise the participation cost is so high that no consumer would be willing to pay any fee to participate in the platform—we have that $\upsilon ^{N}>1$ only if $\epsilon (1-j^{N})-1>0$.

11 Note that Remark (1) can also be interpreted in terms of the elasticity of demand. It states that the lower the demand elasticity (higher $j^{I}$), the higher the optimal fee $\tau ^{I*}$ of the incumbent at the intensive margin.

12 *G* is given by

$$\begin{aligned} G^{w}(w,s^{E})=\left( \left( 1+w\right) ^{-1}-\left( 1-\left( s-s^{E}\right) \right) \left( \left( 1-j^{E}\right) \epsilon -1\right) \right) \left( \left( 1-j^{E}\right) +\frac{1}{\epsilon }\left( \frac{\left( 1+w\right) ^{-1}}{\left( 1-\left( s-s^{E}\right) \right) }-1\right) \right) . \end{aligned}$$

13 Given by

$$\begin{aligned} G^{s}(w,s^{E})=\frac{1}{2\epsilon }\left( \frac{\left( 1+w\right) ^{-1}-\left( 1-\left( s-s^{E}\right) \right) \left( \left( 1-j^{E}\right) \epsilon -1\right) }{\left( 1-\left( s-s^{E}\right) \right) ^{2}}\right) \end{aligned}$$

14 See Appendix (A) for a formal derivation of all the results in this Section.

15 For a search cost $s^{E}$ and intermediation cost *w* low enough, the entrant could find it optimal to lower fees further and attract a consumer set larger than its own good varieties. We abstract from this possibility to simplify the following presentation, as by allowing for it would yield the same main results. The formal assumption for this option to be optimal for the entrant is that $\left( 1-\left( s-s^{E}\right) \right) \left( 1+w\right) >\left( 1+\epsilon j^{n}\right) ^{-1}$.

16 Similar conclusions are obtained when looking at a similar 2019 survey among Polish e-commerce firms by Kantar and Divante consulting, “*The eCommerce Trends Report 2021*”.

17 For Germany: *Gesetz gegen Wettbewerbsbeschränkungen *(GWB). The Act was published in January 2021. For a more extensive discussion on this point, see the sections “M&A activity of the main digital platform providers” and “Amending German competition law for digital regulation” on ‘The Digital Regulation Platform’ (World Bank & ITU); https://digitalregulation.org/.
